# Hybrid Sol–Gel Silica Coatings Containing Graphene Nanosheets for Improving the Corrosion Protection of AA2024-T3

**DOI:** 10.3390/nano10061050

**Published:** 2020-05-29

**Authors:** Nasima Afsharimani, Alicia Durán, Dušan Galusek, Yolanda Castro

**Affiliations:** 1 Department of Coating Processes, FunGlass, Alexander Dubček University of Trenčín, Študentská 2, 91150 Trenčín, Slovakia; dusan.galusek@tnuni.sk; 2 Instituto de Cerámica y Vidrio (CSIC), Campus de Cantoblanco, 28049 Madrid, Spain; aduran@icv.csic.es (A.D.); castro@icv.csic.es (Y.C.); 3 Joint Glass Centre of the IIC SAS, TnUAD and FChPT STU, 91150 Trenčín, Slovakia

**Keywords:** aluminium alloy, sol–gel coatings, corrosion protection, SiO_2_ nanoparticles, graphene nanosheets

## Abstract

In the present work, nanostructured graphene nanosheets were added to hybrid silica sols and deposited on aluminium alloy A2024-T3 to study the effect on the corrosion behaviour. Sols were prepared using tetraethyl-orthosilicate (TEOS), 3-glycidoxypropyl-trimethoxysilane (GPTMS) and a colloidal silica suspension (LUDOX) as silica precursors with adding chemically modified graphene nanosheets (GN-chem). The graphene nanosheets were modified through a straightforward and simple hydrothermal approach and then, dispersed into a silica sol (SiO_2_/GN-chem). ATR-FTIR was used to optimize the silica sol–gel synthesis and to confirm the cross-linking of the silica network. The corrosion behaviour of the SiO_2_/GN-chem coatings was also analysed by electrochemical measurement (potentiodynamic polarization) in 0.05 M NaCl solution. The results showed that the incorporation of modified graphene nanosheets into hybrid silica sol–gel coatings affected the corrosion properties of the substrates. An improvement in the corrosion resistance was observed likely due to the enhanced barrier property and hydrophobic behaviour obtained by incorporation of GN-chem and colloidal silica nanoparticles.

## 1. Introduction

Aluminium alloys such as AA2024-T3 and AA7075-T6 are mainly used in aircraft and automotive industries due to their excellent strength to weight ratio. However, these alloys are susceptible to localized corrosion such as pitting and intergranular corrosion [1,2,3]. Silica-based sol–gel coatings deposited on aluminium substrates have demonstrated good adhesion and corrosion protection performance through forming stable Si-O-Al chemical bonds between the silica layer and the substrate [4].

Over the last few years, graphene appeared as a promising anticorrosion agent for protecting metallic substrates; its impermeability to oxygen and water provides effective barrier properties against the diffusion of corrosion species [5]. Nevertheless, the incorporation of graphene into protective systems has some difficulties due to the poor dispersibility in organic and inorganic solvents. Furthermore, the chemical inertness of graphene prevents its interaction with polymer matrices and aggregates are often formed when graphene is included as nano-filler in the composite systems [6,7,8]. The incorporation of graphene oxide (GO) nanosheets in coatings for enhancing the corrosion properties has been studied in recent years. Liu et al. [9] reported the preparation of thin graphene coatings on aluminium alloys by direct deposition of GO from aqueous solution with effective anticorrosion barrier properties. In another study, W. Sun et al. [10] prepared coatings using (3-aminopropyl)-triethoxysilane (APTES) functionalized with GO nanosheets. Due to the flake-like structure and high aspect ratio of GO sheets, the penetration of the electrolyte was reduced, resulting in good corrosion properties. Ramezanzadeh et al., [11] reported on the synthesis and characterization of silica nanoparticles-covered graphene oxide (SiO_2_-GO) nanosheets and embedded them into epoxy coatings. The results showed that the SiO_2_-GO nano-hybrid coatings significantly reduced the cathodic delamination rate of the epoxy coating, improving the corrosion resistance. More recently, Bing Xue et al. [12] reported the preparation of GO-based hybrid sol–gel coatings on aluminium alloys. In their study, 3-glycidoxypropyl-trimethoxysilane (GPTMS) and zirconium (IV) n-propoxide (TPOZ) were used as precursors and GO was synthesized by oxidizing and exfoliating graphite following a modified Hummers’ method. The characterization showed that C-O-Si bonds formed, enhancing the cross-linking density of the network and improving the corrosion protection of aluminium alloys.

However, above studies focus on direct integration of GO nanosheets to the protective coatings because of the good dispersibility of GO in water and in some organic solvents; yet the preparation of GO follows complicated procedures with successive purification steps that prolong the overall production time and cost. The possibility of direct use of graphene nanosheets would reduce the cost of the procedure, but it is necessary to develop a method for increasing the reactivity of the GN with the sol precursors through introducing functional groups on the surface of GN.

The aim of this work was to use cost-effective large-scale available graphene nanosheets (GN) for incorporation into silica based sol–gel coatings to improve the corrosion protective properties of aluminium alloys (AA2024-T3). Using a simple hydrothermal method, the surface of GN was chemically modified and a visible enhancement of dispersion quality of GN in the silica sol was confirmed. Two types of sols were synthesized based on GPTMS-TEOS (GT) and GPTMS-TEOS-colloidal silica nanoparticles (GTS), respectively. Chemically modified GN were embedded in both sols and deposited on A2024-T3 substrates. Higher quality and better protective coatings were achieved in the case of GTS system with chemically modified GN. This effect could be attributed to the incorporation of colloidal silica nanoparticles together with addition of GN that increased the density of silica coatings and decreased the hydrophilic property.

## 2. Materials and Methods

### 2.1. Substrate Preparation

Aluminium alloy AA2024-T3 (Cu 4.9 wt.%, Mg 1.28 wt.%, Mn 0.629 wt.%, Ti 0.0154 wt.%, Zn 0.142 wt.%, Fe 0.239 wt.%, the remaining Al) with dimensions 40 × 20 × 1 mm, was used as substrate. The alloys were pre-cleaned using an alkaline cleaning solution (Metaclean T2001—Chemie Vertrieb Hannover GmbH & Co Hannover KG, Hannover, Germany), an alkaline etching solution (Turco Liquid Aluminetch Nr.2—Turco Chemie, Hamburgo GmbH, Hamburg, Germany) and, finally, an acid etching solution (Turco Liquid Smutgo NC—Turco Chemie Hamburgo GmbH, Hamburg, Germany). This procedure enabled to remove the native oxide layer and intermetallic particles on the metal surface. Before the deposition, samples were rinsed with deionized water and dried in air.

### 2.2. Chemical Modification and Characterization of Graphene Nanosheets

Graphene nanosheets were chemically modified mixing 1 g of GN powder (GRAPHENIT-OX from Nanoinnova Technologies SL, Toledo, Castilla la Mancha, Spain, less than 5 layers, average flake size of 1–3 micrometre) with distilled water (H_2_O, 100 mL), phosphoric acid (H_3_PO_4_, 5 mL) and ammonium persulfate ((NH_4_)_2_S_2_O_8_, 0.5 g). The solution was sonicated for 1 h using an ultrasound probe (Ultrasonication Probe, GM 2200, Bandelin electronic, Berlin, Germany). Then, the solution was transferred to a hydrothermal reactor and kept at 150 °C for 24 h. After cooling down to the room temperature, the obtained chemically modified GN (GN-chem) were separated by filtration and washed several times using deionized water and a final step with ethanol to reach at pH = 6–7. Finally, GN-chem were dried in an oven at 50 °C for 18 h and stored.

GN-chem nanosheets were characterized by Fourier transform Infrared (FTIR) and Raman spectroscopies. FTIR spectra were recorded between 650 and 4000 cm^−1^ with a resolution of 2 cm^−1^, using a Perkin Elmer Spectrum 100 spectrometer (Madrid, Spain) with an Attenuated Total Reflectance (ATR) accessory of diamond/ZnSe crystal. Spectra were collected from 4000 cm^−1^ to 650 cm^−1^ and were processed and plotted as log of the inverse reflectance, log (1/R), which is equivalent, to the absorbance (A).

Raman spectra were acquired with a Raman-AFM microscope (Alpha 300 AR, WITec, Ulm Germany) between 450 and 3200 cm^−1^ with a resolution of 2 cm^−1^. An Nd:YAG laser, emitting at a wavelength of 532 nm, was used as the source of excitation and the power was about 4 mW. The acquisition time and the accumulation number were set to 10-second exposure and 3 accumulations, respectively.

### 2.3. Synthesis and Characterization of Hybrid Silica Sols

Alkoxide precursors and other reagents were used as received, without further purification. Different silica and GN-silica sols were developed. First, GN or GN-chem were dispersed in tetrahydrofuran (THF) and sonicated for 15 min to obtain a stable suspension of 25 mg/mL concentration. Then, a small amount of 3-glycidolxypropyl trimethoxysilane (GPTMS, ABCR, 98%) (160 µL) was added to the suspensions and maintained under stirring for 6 h at room temperature and 1 h more at 85 °C. The resulting suspensions were labelled as GN/THF/GPTMS and GN-chem/THF/GPTMS, respectively, and were further incorporated in two different hybrid silica sols.

The first silica sol (total volume: ~55 mL) was prepared by mixing tetraethoxysilane (TEOS, Aldrich-Sigma, Madrid, Spain 99%) and GPTMS with absolute ethanol (EtOH, Panreac, Castellar del Vallès (Barcelona) Spain 99.8%) with mole ratios of 0.05TEOS:0.05 GPTMS: 0.40 EtOH. The sol was maintained under stirring for 15 min at room temperature. Then, 1.1 mL of GN/THF/GPTMS or GN-chem/THF/GPTMS was added. Finally, acidulated water (HCl 1 M, 6.7 mL) was added drop by drop to the solutions under constant stirring and maintained at 40 °C for 24 h. The resultant sols (denoted as GT, GT/GN and GT/GN-chem) were aged during 24 h before being used (Table 1).

The second type of silica sols (total volume: ~53 mL), denoted as GTS and GTS/GN-chem, were prepared incorporating a colloidal silica nano-particles suspension (LUDOX, 40 wt.%, 20 nm, pH = 9 Aldrich, Madrid, Spain). GPTMS, TEOS and LUDOX were mixed with mole ratios of 0.05:0.05:0.14, respectively. The mixture was stirred for 5 min and then, concentrated HNO_3_ (VWR, 65%, 0.32 mL) was added for catalysing the reactions. On the other hand, GTS/GN-chem sol was prepared following the same procedure that GTS; GN-chem nanosheets (0.026 g) were directly incorporated to GPTMS/TEOS/colloidal silica sol before the addition of the nitric acid. Finally, EtOH was added to GTS and GTS/GN-chem sols to reach a final SiO_2_ concentration of 262 g/L.

Table 1 shows the different compositions prepared together with the mole ratios and silica concentration. The final concentration of GN and GN-chem in the sols was 0.5 mg/mL.

The evolution of the sols was followed by ATR-FTIR spectroscopy to identify the hydrolysis and condensation reactions. ATR-FTIR spectra were recorded between 650 and 4000 cm^−1^, using a Perkin Elmer Spectrum 100 spectrometer (Madrid, Spain) with an Attenuated Total Reflectance (ATR) accessory, with a resolution of 2 cm^−1^. UV–vis-NIR spectra were recorded between 2500 and 1800 nm with a Perkin Elmer (Lambda 950) spectrometer (Madrid, Spain), with a resolution of 2 nm. The viscosity of the sols was measured using an A & D Vibro Viscometer SV-10 (Oxfordshire, United Kingdom) as a function of time to evaluate the stability of the sols.

### 2.4. Deposition and Characterization of Hybrid Silica Sol–Gel Coatings

Considering the coating preparation, the sols with better dispersibility of graphene nanosheets and stability were selected for coating preparation. GT, GT/GN-chem, GTS and GTS/GN-chem sols were deposited on glass slides (soda-lime microscope slides, Menzel-Glaser) and on AA2024-T3 substrates. The deposition was carried out by dip-coating at withdrawal rate of 30 cm/min. Bilayer coatings were prepared by a multistep deposition using a dry step of 25 °C for 5 min. All samples were thermally treated at 120 °C for 1 h using an electric oven (Memmert UE55).

The coating thickness was measured on coated glass slides using Spectroscopic Ellipsometer (J.A.Woollam Co., Inc, Lincoln, NE, USA, EC-400, M-2000U Software: WVASE32). The spectral bands were recorded from 250 to 900 nm at incident angles between 50° and 60°. The data were fitted using the WVASE32 software with a Cauchy model.

Water contact angle measurements were also performed to determine the hydrophilic/hydrophobic behaviour of the films using the Easy Drop Standard ‘Drop Shape Analysis System’ Kruss DSA 100 equipment under ambient laboratory conditions. Three measurements were performed on each sample and the results are presented as average ± standard deviation.

The surface morphology and cross-section view of the samples were observed by atomic force microscopy (AFM, CERVANTES NANOTEC Electronic, Madrid, Spain), and scan electron microscopy (SEM, HITACHI TM1000), respectively. For SEM characterization, the samples were prepared by bending the samples over 180° to observe the cross-section and the adhesion between the coating and the substrate. Additionally, adhesion test was carried out using the Scotch Tape test. The test was performed applying the tape and pressuring on an area of the coating. Adhesion is considered adequate in case the coating is not pulled off by the tape when it is removed from the sample.

The corrosion properties of coated samples were evaluated by potentiodynamic polarization measurements in 0.05 M NaCl aqueous solution at room temperature using a Gamry FAS2 Femtostat (Warminster, PA, USA) and a typical three-electrode configuration. A saturated calomel electrode (SCE) was used as the reference electrode, and a platinum wire as the counter electrode. The sample acted as the working electrode (exposed area of 0.79 cm^2^). Prior to each polarization test the samples were immersed in the electrolyte for 1 h to reach the equilibrium. Potentiodynamic polarization curves were recorded starting at −500 mV vs. *E*_OC_ up to 1 V at a scan rate of 1 mV/s. Experiments were repeated at least three times and the most representative measurements were indicated. The corrosion potential (*E*_corr_) and corrosion current density (*j*_corr_) were determined.

## 3. Results and Discussion

The chemical modification of GN was studied by ATR-FTIR and Raman spectroscopies. The sol–gel synthesis and chemical reactions were followed by ATR-FTIR and UV–vis-NIR spectroscopy and by viscometer. The stability of the sols was followed by viscometry and the coatings were characterized by measuring the thickness, contact angle and electrochemical test.

### 3.1. Characterization of Modified Graphene Nanosheets

Graphene nanosheets (GN) were chemically modified according to the above-mentioned hydrothermal procedure. The FTIR spectra of GN and chemically modified GN (GN-chem) are presented in Figure S1 (supporting information) [13]. In the spectrum of GN, the appeared bands are associated with –OH (~3000–3250 cm^−1^), –CH_2_ (~2850–2923 cm^−1^), C=C (~1580–1630 cm^−1^) and C–O–C (~1225 cm^−1^) groups, indicating the presence of surface hydroxyl groups and also adsorbed water molecules [11,14], symmetric and asymmetric C–H bands in CH_2_ [14,15], C skeletal vibrations [14] and epoxy [14,16] groups, respectively. After chemical modification (GN-chem) the FTIR spectrum changed. The peaks corresponding to hydroxyl groups (–OH) and symmetric and asymmetric C–H bands in CH_2_ were disappeared. Further, the band at ~1580 cm^−1^ associated with C=C skeletal vibrations increased in intensity [11,17,18] and a new band at ~1000 cm^−1^ associated to C–O–C epoxy groups appeared. The presence of C=C aromatic band indicated the stability of the graphene structure even after the chemical modification. In addition, the emergence of the new band suggested the presence of oxygen atoms or cyclic ether accumulated at the edges of defects in GN-chem [19]. The oxygen-containing groups in the spectrum revealed the successful conversion of inert GN into modified GN-chem. It is worth mentioning that, the slight deformation of the red spectrum with the sharp band around 1600 cm^−1^ could be the result of the high refractive index of our material and its strong absorption. Therefore, in order to omit such effect, it is recommended to replace the diamond accessory, used in this study, by a Ge plate that has a higher refractive index, close to our carbon material. However, the authors have used ATR for qualitative analysis to be completed by Raman. In general, the type of the crystal accessory should not necessarily affect the position of the bands. Moreover, both Raman and ATR techniques led to comparable results demonstrating the suitability of using them together.

**Figure 1:**
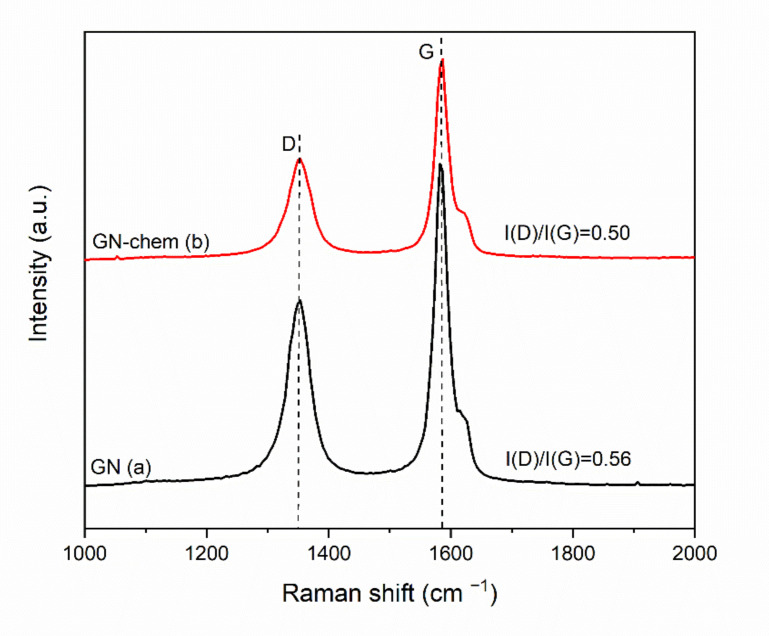
Raman spectra of (**a**) pristine graphene nanosheets (GN) and (**b**) chemically modified graphene nanosheets (GN-chem).

Raman scattering spectroscopy was used to explore the structural properties and to study the disorder and defects in crystal structure. Raman spectra of GN and GN-chem are shown in Figure 1. In both samples, Raman spectra showed D and G bands at 1351 cm^−1^ and 1584 cm^−1^, respectively. The G band corresponds to sp^2^ carbon forms, and is associated with the C–C bond stretch. This band is formed from first order Raman scattering [20].

The D band arises from the zone boundary phonons and is attributed either to various defects or to a breakdown of translational symmetry in graphite lattice [21]. Disorder is determined by the width of the D band (FWHM). Typically, the broader the D band (higher values of FWHM), the smaller the size of the sp^2^ domains being associated with the vacancies and distortions during oxidation or chemical processes [22]. The D and G bands were fitted to a Lorentzian function and the FWHM of the D band in GN-chem was determined to be around 43.02 cm^−1^ while the FWHM in GN was 44.19 cm^−1^. The slight narrowing down the linewidth of the D band in GN-chem could be the result of the increased in-plane crystallite size by partial restoration of sp^2^ carbon domains [23]. This might further be supported by the ratio of the intensity of the G band to that of the D band which is also related to the in-plane crystallite size, La. The in-plane crystallite sizes of GN and GN-chem, which were calculated using the relationship La (nm) = 4.4/(I(D)/I(G)) [23] were about 7.85 nm and 8.80 nm, respectively, for which the corresponding I(D)/I(G) ratios were 0.56 and 0.5, respectively. Apparently, the chemical modification process enabled the restoration of sp^2^ domains and aromatic structure of carbon atoms.

**Figure 2:**
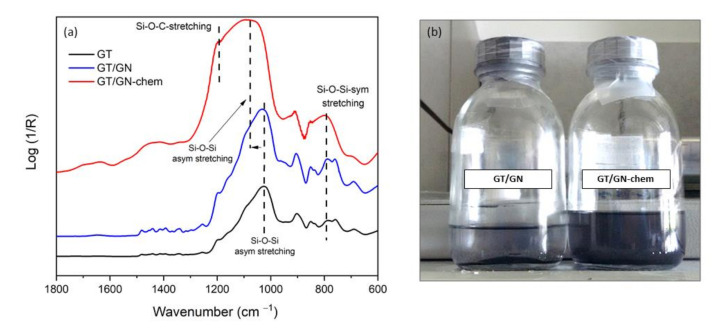
FTIR spectra of 3-glycidoxypropyl-trimethoxysilane (GPTMS)-tetraethyl-orthosilicate (TEOS) (GT), GT/GN and GT/GN-chem (**a**) and visual image of the stability of GN and GN-chem nanosheets in GT sols after 72 h (**b**).

Therefore, considering both FTIR and Raman analyses, it is likely that chemical modification of GN nanosheets firstly, led to elimination of some groups (hydroxyl and R1, R2 = –CH_2_, –CH_3_) from the edge and the basal plane of the sheets resulting in partial restoration of sp^2^ carbon domains. Such modification might have also helped the GN-chem nanosheets to act more efficiently as “physical barriers” in hybrid coatings while exposed to the corrosive environment. In addition, secondly, the appearance and intensification of some vibrational bands such as C–O–C in FTIR results suggest that some reactive groups such as epoxy were created during the chemical modification. All this might be the reason for the improved dispersability and stability of silica sols based on GN-chem compared to GN. An schematic illustration of chemical modification of GN nanosheets and possible changes in functional groups during the modification process is shown in Figure S2. It is worth mentioning that, although the pristine GN nanosheets included some oxygen functionalities, the silica sols synthesized with them showed poor stability (Figure 2b) making them inappropriate for coating fabrication. The chemical modification proposed in our study, could have destroyed parts of the hydroxyl groups, resulting in partial restoration of sp^2^ carbon atoms (as observed in Raman spectra and schemed in Figure S2) which in principle appeared to work better in condensation with siloxane precursors.

### 3.2. Synthesis and Characterization of the Sols

In order to analyse the chemical crosslinking of GN into inorganic-organic hybrid silica sols, FTIR spectra of pure GPTMS-TEOS sols (GT), GT sols containing GN (GT/GN) and GN-chem nanosheets (GT/GN-chem) are presented in Figure 2. The spectra of all samples included absorption bands at 1040–1080 cm^−1^ and 800 cm^−1^ assigned to asymmetric and symmetric stretching of Si-O-Si network, respectively [16,24]. In the case of GT/GN-chem, these bands broadened considerably and the band at around 1040–1080 cm^−1^ shifted towards larger wavenumbers most likely due to the chemical bonding between the GN-chem sheets and the silica network. The functional groups on GN-chem were expected to react with the hydrolysed species of TEOS and GPTMS molecules. Furthermore, a new band appeared at around 1205 cm^−1^ assigned to stretching of Si-O-C network [25]. These bands confirmed the cross-linking between GN-chem and siloxane network structure. Moreover, the disappearance of absorption band at 1250 cm^−1^ (ring breathing) and weakening of the band intensity at around 910 cm^−1^ (asymmetric stretching) of epoxy rings of GPTMS [26] also revealed the cross-linking between the functional groups on GN-chem and the GPTMS.

In the case of GT/GN sol, the asymmetric stretching of Si-O-Si network was confirmed by the band at ca. 1040–1080 cm^−1^; Si-O-C band was not observed in this system. Figure 2b represents a visual image of the GT/GN and GT/GN-chem sols, confirming the better dispersibility of GN-chem compared to GN and the cross-linking between GN-chem and Si-O-Si network.

Furthermore, UV–vis-NIR absorption measurement was carried out on GT and GT/GN-chem shown in Figure S3. The broad band between 1950 and 2175 nm in the spectrum of GPTMS assigned to epoxy rings [27,28] was partially disappeared in GT and GT/GN-chem sols that might indicate the opening of some of the epoxy rings, agreeing with the FTIR analysis. The polymerization continued during the synthesis, in the liquid state, resulting in a more cross-linked Si-O-Si network and, consequently, led to a more integrated coating. However, in order to give a deeper interpretation of UV absorption data, especially for GT/GN-chem, further studies are required.

In addition, the viscosity of GT, GT/GN-chem, GTS and GTS/GN-chem sols was measured at room temperature being 3.2 ± 0.4, 3.4 ± 0.2, 3.8 ± 0.2 and 3.9 ± 0.3 mPas.s, respectively. The low viscosity values permitted obtaining homogeneous coatings by dipping. The stability of sols, evaluated following the viscosity as a function of time, was convenient, maintaining the values for at least 2 weeks.

### 3.3. Characterization of the Coatings

Homogeneous and transparent coatings were obtained for all the sols. The thickness of bilayer GT, GT/GN-chem, GTS and GTS/GN-chem coatings deposited on glass slides and cured at 120 °C was measured by ellipsometry (Table 2). Thicker coatings were obtained for GTS and GTS/GN-chem systems associated with the incorporation of SiO_2_ nanoparticles. In the case of GT and GT/GN-chem coatings, thicknesses in the range of 2.1 ± 0.7 μm and 2.3 ± 0.6 μm were measured, respectively, while for GTS and GTS/GN-chem the thickness increased up to 5.3 ± 0.3 μm and 5.5 ± 0.2 μm, respectively.

**Figure 3:**
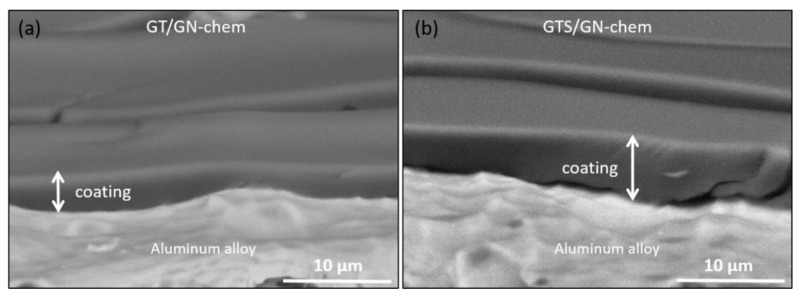
SEM images of cross sections of GT/GN-chem (**a**) and GTS/GN-chem coatings (**b**) on AA2024 alloy.

Figure 3 shows the SEM images of the cross sections of GT/GN-chem and GTS/GN-chem coatings onAA2024. As it was observed, AA2024 substrates were completely covered by the films and the thickness values were in agreement with the values measured by Ellipsometry. Moreover, the films were well adhered to the surface of substrates. The cracks observed in the coatings appeared during the sample preparation for SEM analysis. In addition, Scotch Tape test confirmed the suitable adhesion of the coatings. In addition, AFM topography images in Figures S4 and S5 show surface morphological modifications of the coatings. The surface roughness values are presented in Table 2. The surface roughness increased from 0.5 nm and 2.3 nm for GT and GTS coatings to 0.9 nm and 3.6 nm for GT/GN-chem and GTS/GN-chem, respectively.

Contact angles of coatings on AA2024 are summarized in Table 2. All the coatings showed contact angles between 55° and 74°. The addition of GN-chem increased the hydrophobicity of the layers, GT/GN-chem and GTS/GN-chem coatings showed higher contact angles compared to GT and GTS.

Electrochemical properties of bare AA2024-T3 substrates and coated (GT, GT/GN-chem, GTS and GTS/GN-chem) substrates were studied by potentiodynamic measurements in 0.05 M NaCl. The effect of coating composition through the addition of colloidal silica nanoparticles and graphene nanosheets on the barrier behaviour was explored.

**Figure 4:**
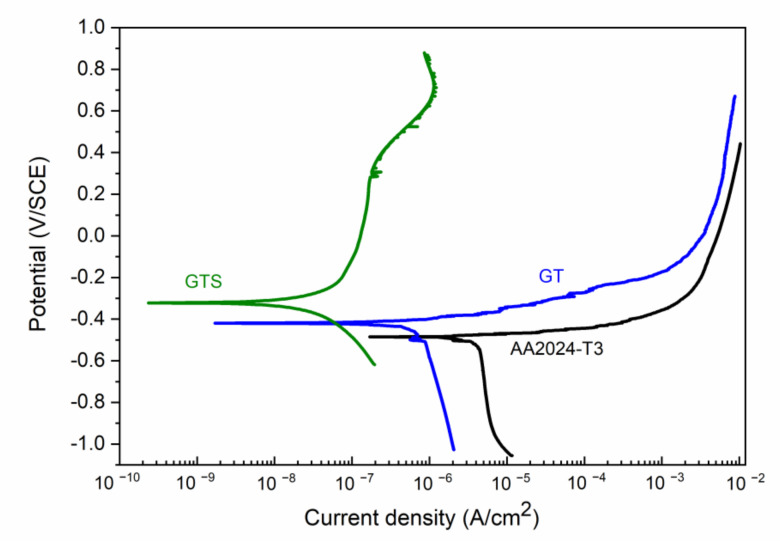
Potentiodynamic polarization curves of bare AA2024-T3 alloy and substrates coated with hybrid sol–gel coatings without and with colloidal SiO_2_ nanoparticles denoted as GT and GTS, respectively.

Figure 4 shows the polarization curves of bare AA2024-T3 compared to GT and GTS coatings. The bare AA2024-T3 immersed in chloride solution is susceptible to localized corrosion (black curve in Figure 4). In the cathodic range, oxygen reduction takes place, while in the anodic range the corrosion starts above *E_corr_*. In GT coatings, corrosion current density (*j_corr_* = 0.5 ± 0.1 µA/cm^2^) decreased one order of magnitude with respect to bare AA2024 alloy (*j_corr_* = 3.0 ± 0.5 µA/cm^2^) while the *E_corr_* was maintained at around −0.42 ± 0.02 V. In the case of GTS coating, better corrosion was observed compared to GT coatings, showing a smaller corrosion current density (j_corr_ = 0.03 ± 0.01 µA/cm^2^) and a broad passive range (or barrier) with a passivation current (*j_p_*) of ca. 0.2 ± 0.1 µA/cm^2^ (*ΔE* in Table 3). Furthermore, *E_corr_* was shifted to −0.31 ± 0.03 V. The presence of silica nanoparticles allowed the increase of the density and the thickness of the coatings alongside with the cross-linking of the silica network [29,30]. All this led to better corrosion resistance of GTS coatings with respect to AA2024 substrate.

**Figure 5:**
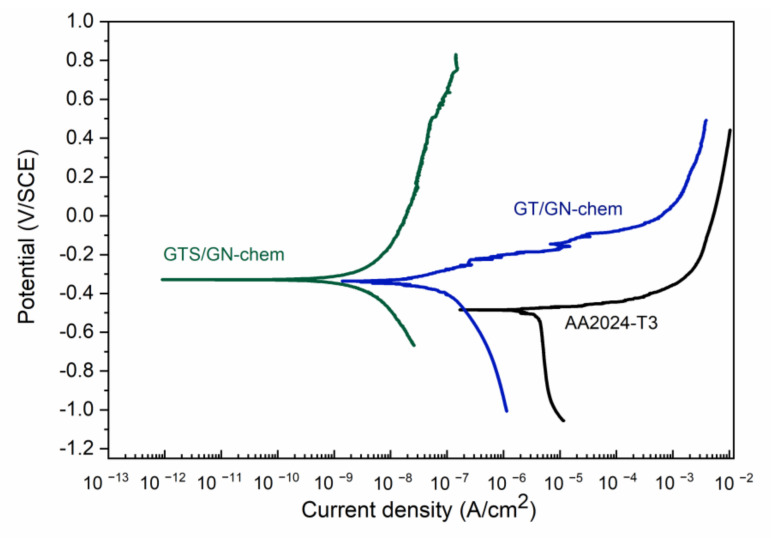
Potentiodynamic polarization curves of bare AA2024-T3 and substrates coated with hybrid sol–gel coatings including chemically modified graphene nanosheets.

Figure 5 shows the polarization curves of bare AA2024-T3, GT/GN-chem and GTS/GN-chem coatings. After addition of graphene nanosheets, the coatings revealed positive shifts in *E_corr_* and decreases in corrosion current densities with respect to bare AA2024-T3. Comparing the GT (Figure 4) and GT/GN-chem coatings (Figure 5), *E_corr_* shifted from −0.42 V to −0.33 V, respectively. The corrosion current density decreased down to *j_corr_*= 0.06 ± 0.01 µA/cm^2^. According to water contact angle measurements (Table 2), the addition of graphene nanosheets increased the hydrophobicity of GT/GN-chem coatings, which positively affected the corrosion behaviour. Table 3 shows electrochemical corrosion parameters extracted from Figure 4 and Figure 5.

A more interesting behaviour was observed for GTS/GN-chem coatings, where a significant decrease in corrosion current density (*j_corr_* = 0.002 ± 0.001 µA/cm^2^) was observed together with the presence of a pseudo-passive region indicating that anodic reaction had been inhibited. The *E_corr_* stayed around −0.32 V, similar to the value of pure GTS coatings.

The surface morphology, the wettability and the structural integrity of the coatings play a key role in the corrosion behaviour. This means that hybrid coatings with well-integrated structure, lower wettability (higher hydrophobicity), larger thickness and higher roughness are good candidates for corrosion protection applications.

According to previous studies, the inclusion of fillers such as graphene nanosheets and silica nanoparticles into composite matrices increased the surface roughness [31,32]. In the present study, when the modified graphene nanosheets (GN-chem) were embedded into GT and GTS sols, the presence of oxide functionalities and fewer number of stacked layers, resulted from chemical modification/exfoliation and confirmed by FTIR and Raman analyses, increased the surface area and the surface roughness of the corresponding coatings. AFM topography images in Figures S4 and S5 revealed such surface morphological modifications. In addition, the increase in surface roughness led to higher hydrophobicity confirmed by the contact angle measurements of GN-chem based coatings. Since the corrosion mechanism relies on the presence of water to take place, the use of a hydrophobic coating can retard the penetration of water to the underlying metal surface.

On the other hand, the presence of silica nanoparticles increases the cross-linking [29]. These particles can also fill the pores which lead to the barrier effect [30]. When the colloidal silica nanoparticles and GN-chem nanosheets were added into GT sols, the presence of reactive groups on the surface of silica nanoparticles (–OH) and chemically modified graphene nanosheets (–OH and epoxy groups) produced rougher surfaces with larger surface area [33]. The larger surface area could increase the reactivity of the silica nanoparticles and GN-chem nanosheets not only with each other but also with the surrounding matrix.

Regarding the electrochemical analyses, better corrosion properties were observed for GTS coatings compared to GT coatings. The smaller corrosion current density and the more positive corrosion potential of GTS coatings can be associated with more integrated structure formed by the silica nanoparticles. Corrosion behaviour was further improved when GN-chem nanosheets were added into GTS sols. The further enhancement of the corrosion protection property of the GTS/GN-chem coatings, as mentioned above, can be referred to the promoted surface roughness, the higher hydrophobicity and the enhanced cross-linking within the coatings [12,28]. The combination of graphene nanosheets and colloidal SiO_2_ nanoparticles provided a suitable system for improving the corrosion resistance of aluminium alloys. The results revealed that the incorporation of GN-chem nanosheets into silica coatings affects the cathodic (O_2_ + 2H_2_O + 4e → 4OH^−^) as well as anodic (Al → 3e + Al^3+^) behaviour [10,25].

Figure S6 shows a simplified schematic illustration of GT, GT/GN-chem, GTS and GTS/GN-chem coatings. The structure and the network integrity are shown before and after addition of GN-chem and SiO_2_ nanoparticles. In addition, the effectiveness of filler addition to the coatings in achieving an impermeable barrier is shown schematically in Figure S7. According to the illustration, the presence of GN-chem sheets together with SiO_2_ nanoparticles can block the penetration of corrosive ions and retard the corrosion process through establishment of higher cross-linked structure by forming a good physical barrier (passivation regions). Eventually, a visual inspection of the surface coating alteration after exposure to the corrosive environment is shown in Figure S8. As evidenced by the image, the lowest surface damage is observed for the GTS/GN-chem coating.

## 4. Conclusions

Hybrid silica sol–gel coatings containing graphene nanosheets were synthesized to improve the corrosion protection of aluminium alloys AA2024-T3. To the best of our knowledge, this is the first time that the synthesis protocol of silica sols based on introduction of graphene nanosheets with colloidal SiO_2_ nanoparticles is reported. In addition, the study proposes the use of slightly modified graphene nanosheets to silica sol–gels, instead of graphene oxide nanosheets that are costly and include tedious preparation and purification steps.

The chemical modification of graphene nanosheets was carried out through a simple hydrothermal approach and studied by ATR-FTIR and Raman spectroscopies. The chemical modification improved the stability of graphene nanosheets into the sol solutions. According to surface chemical, structural and electrochemical characterizations, incorporation of chemically modified graphene nanosheets into TEOS and GPTMS sols including colloidal SiO_2_ led to the best results. The films based on chemically modified graphene and SiO_2_ nanoparticles (GTS/GN-chem coating) were thicker and with a more hydrophobic surface, having improved the protection of AA2024 substrates.

The corrosion behaviour evaluated by potentiodynamic revealed good corrosion property for the GTS/GN-chem system. The corrosion current density of the GTS/GN-chem system significantly decreased with respect to the GT coating and bare AA2024 substrate, showing a passivation range. Such barrier properties are associated with the chemical interactions between the coating and the substrate and with the increment of cross-linking of GN-chem based coatings.

These coatings could be promising candidates to be applied on the surface of AA2024-T3 alloys combined with the deposition of an organic top coat.

## Figures and Tables

**Table 1:** Compositions and mole ratios of the precursors used to prepare different hybrid silica sols with and without graphene nanosheets.

Sample	GN (g)	GPTMS (mol)	TEOS (mol)	EtOH (mol)	HCl (1M) (mol)	HNO_3_ (mol)	LUDOX (mol)	SiO_2_ Concentration (g/L)
**GT**	_	0.05	0.05	0.40	0.38	_	_	109
**GT/GN**	0.027	0.05	0.05	0.40	0.38	_	_	109
**GT/GN-chem**	0.027	0.05	0.05	0.40	0.38	_	_	109
**GTS**	_	0.05	0.05	0.40	_	0.01	0.14	262
**GTS/GN-chem**	0.026	0.05	0.05	0.40	_	0.01	0.14	262

**Table 2:** Thickness, water contact angles and surface roughness of GT, GT/GN-chem, GPTMS-TEOS-colloidal silica (GTS) and GTS/GN-chem coatings.

Coating	Thickness (μm)	Contact Angle (°)	Roughness (nm)
**GT**	2.1 ± 0.7	61 ± 4	0.5 ± 0.1
**GT/GN-chem**	2.3 ± 0.6	72 ± 2	0.9 ± 0.2
**GTS**	5.3 ± 0.3	55 ± 2	2.3 ± 0.2
**GTS/GN-chem**	5.5 ± 0.2	74 ± 2	3.6 ± 0.1

**Table 3:** Electrochemical parameters: corrosion potential (*E_corr_*), corrosion current density (*j_corr_*), passivation current (*j_p_*) and passive region (*ΔE*) of bare AA2024-T3 and substrates coated with sol–gel coatings with and without graphene nanosheets. The parameters determined from potentiodynamic polarization curves (Figure 4 and Figure 5).

Sample	*E_corr_* (V)	*j_corr_* (µA/cm^2^)	*j_p_* (µA/cm^2^)	*ΔE* (V)
**AA2024-T3**	−0.48 ± 0.03	3.0 ± 0.5	_	_
**GT**	−0.42 ± 0.02	0.5 ± 0.1	_	_
**GT/GN-chem**	−0.33 ± 0.03	0.06 ± 0.01	_	_
**GTS**	−0.31 ± 0.03	0.03 ± 0.01	0.2 ± 0.1	0.37
**GTS/GN-chem**	−0.32 ± 0.04	0.002 ± 0.001	0.9 ± 0.1	0.40

## Supplementary Materials

The following are available online at htttp://https://www.mdpi.com/2079-4991/10/6/1050/s1, Figure S1: FTIR spectra of (**a**) pristine graphene nanosheets (GN) and (**b**) chemically modified graphene nanosheets (GN-chem). The band appeared at around 2000 cm ^−1^ in GN might be assigned to (CO, stretch); though further studies are required to elucidate the origin of this peak and the accuracy of the assignment, Figure S2: Schematic illustration of chemical modification of GN nanosheets and possible changes in functional groups during the modification process with respect to FTIR and Raman observations. As shown, elimination of some groups from the edge and the basal plane (hydroxyl and R1, R2 = -CH_2_, -CH_3_) led to partial restoration of SP^2^ domains and aromatic structure of carbon atoms; this might have also helped the GN-chem nanosheets to act more efficiently as “physical barriers” in hybrid coatings while exposed to the corrosive environment. In addition, according to FTIR, appearance and intensification of some vibrational bands such as C-O-C may suggest that some reactive groups such as epoxy were created during the chemical modification, Figure S3: UV-vis-NIR spectra of GPTMS, GT and GT/GN-chem sols, Figure S4: AFM topography images of (**a**) GT, (**b**) GT/GN-chem, (**c**) GTS and (**d**) GTS/GN-chem coatings on aluminium alloy substrates, Figure S5: AFM topography 2D images and corresponding 3D views of surface morphology of (**a**) GT, (**b**) GT/GN-chem, (**c**) GTS and (**d**) GTS/GN-chem coatings on aluminium alloy substrates. (XYZ axis values are (**a**) 2.0 µm, 2.0 µm, 3.4 nm (**b**) 2.0 µm, 2.1 µm, 6.2 nm (**c**) 2.0 µm, 2.1 µm, 15.6 nm and (**d**) 2.0 µm, 2.0 µm, 41.8 nm, respectively, Figure S6: Schematic illustration of GT, GT/GN-chem, GTS and GTS/GN-chem coatings. The structure and the network integrity are shown before and after addition of fillers (GN-chem and SiO_2_ nanoparticles), Figure S7: Schematic illustration of the effectiveness of filler addition (GN-chem and SiO_2_ nanoparticles) to the coatings in achieving an impermeable barrier against the penetration of corrosive ions. (Red arrows show the track of corrosive ions attacking and penetrating into the coating.), Figure S8: Visual illustration of the surface coating alteration after exposure to the corrosive environment. As evidenced by the image, the lowest surface damage is observed for the GTS/GN-chem coating.
