# Supplementary material for: Hybrid Sol–Gel Silica Coatings Containing Graphene Nanosheets for Improving the Corrosion Protection of AA2024-T3
Source: Nanomaterials (Basel). 2020 May 29;10(6):1050. doi: 10.3390/nano10061050 (PMC7352741; doi:10.3390/nano10061050)
Supplement: Supplementary file 1 [file nanomaterials-10-01050-s001.pdf]

nanomaterials

Supplementary Materials

Hybrid Sol–Gel Silica Coatings Containing Graphene Nanosheets for Improving the Corrosion Protection of AA2024-T3

Nasima Afsharimani [1,*], Alicia Durán [2], Dušan Galusek [1,3] and Yolanda Castro [2]

1 Department of Coating Processes, FunGlass, Alexander Dubček University of Trenčín, Študentská 2, 91150 Trenčín, Slovakia; dusan.galusek@tnuni.sk
2 Instituto de Cerámica y Vidrio (CSIC), Campus de Cantoblanco, 28049 Madrid, Spain; aduran@icv.csic.es (A.D.); castro@icv.csic.es (Y.C.)
3 Joint Glass Centre of the IIC SAS, TnUAD and FChPT STU, 91150 Trenčín, Slovakia
* Correspondence: nasima.afsharimani@tnuni.sk

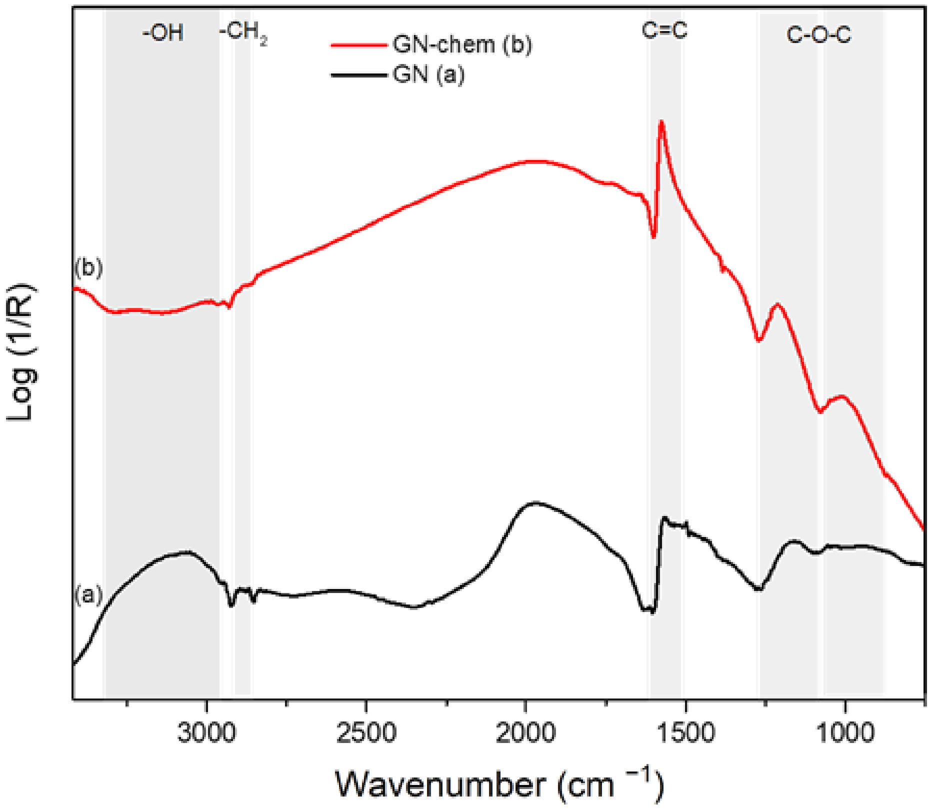

Figure S1. FTIR spectra of (**a**) pristine graphene nanosheets (GN) and (**b**) chemically modified graphene nanosheets (GN-chem). The band appeared at around 2000 cm⁻¹ in GN might be assigned to (CO, stretch)[1]; though further studies are required to elucidate the origin of this peak and the accuracy of the assignment.

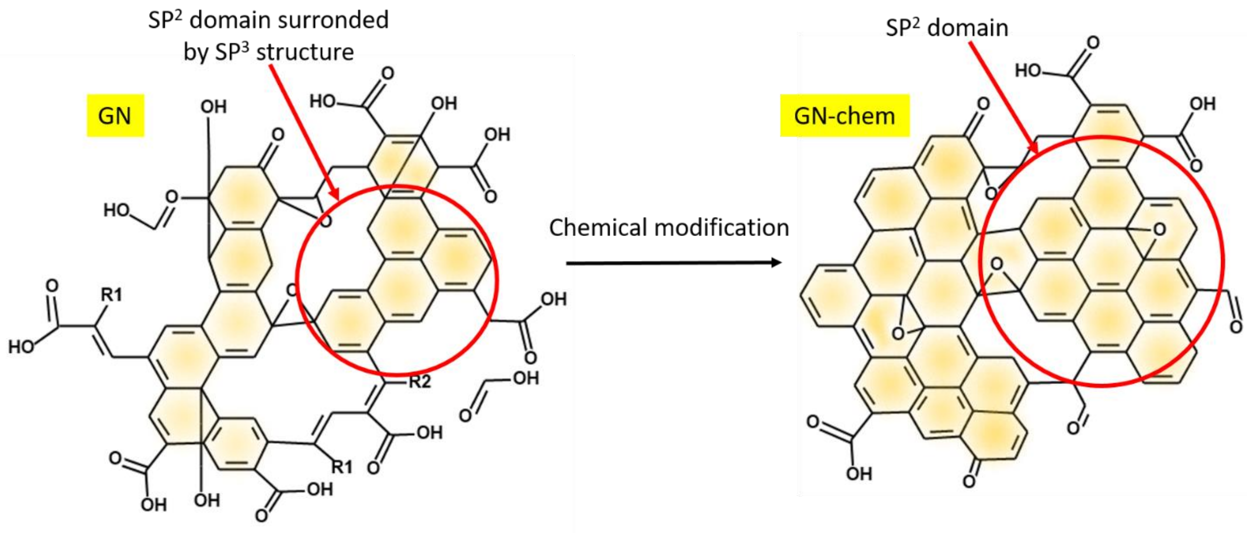

Figure S2. Schematic illustration of chemical modification of GN nanosheets and possible changes in functional groups during the modification process with respect to FTIR and Raman observations. As shown, elimination of some groups from the edge and the basal plane (hydroxyl and R1, R2 = -CH_2, -CH_3) led to partial restoration of SP^2 domains and aromatic structure of carbon atoms; this might have also helped the GN-chem nanosheets to act more efficiently as "physical barriers" in hybrid coatings while exposed to the corrosive environment. In addition, according to FTIR, appearance and intensification of some vibrational bands such as C–O–C may suggest that some reactive groups such as epoxy were created during the chemical modification.

The UV-vis-NIR absorption spectra of GPTMS, GT and GT/GN-chem sols are presented in Figure S3. As underlined in the manuscript, the broad peak between 1950 and 2175 nm in the spectrum of GPTMS is assigned to epoxy rings. These bands partially disappeared in GT and GT/GN-chem sols might indicating the opening of some of the epoxy rings, agreeing with the FTIR analysis, and resulted in a more cross-linked Si–O–Si network.

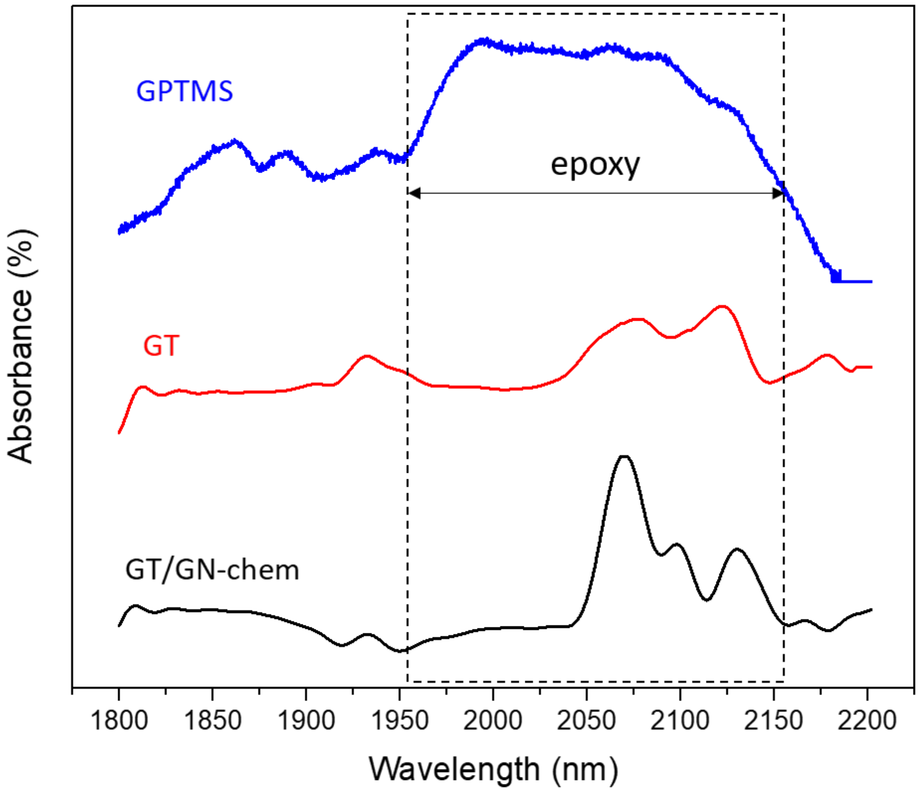

Figure S3. UV-vis-NIR spectra of GPTMS, GT and GT/GN-chem sols.

The surface morphology of GT, GT/GN-chem, GTS and GTS/GN-chem coatings were investigated by AFM as depicted in Figure S4. Smoother surfaces were observed for GT and GTS samples. According to roughness analysis in Figure S5, the surface roughness increased from 0.5 nm and 2.3 nm for GT and GTS coatings to 0.9 nm and 3.6 nm for GT/GN-chem and GTS/GN-chem, respectively. It is known that the inclusion of fillers such as graphene nanosheets and silica nanoparticles into composite matrices increase the surface roughness [2,3].

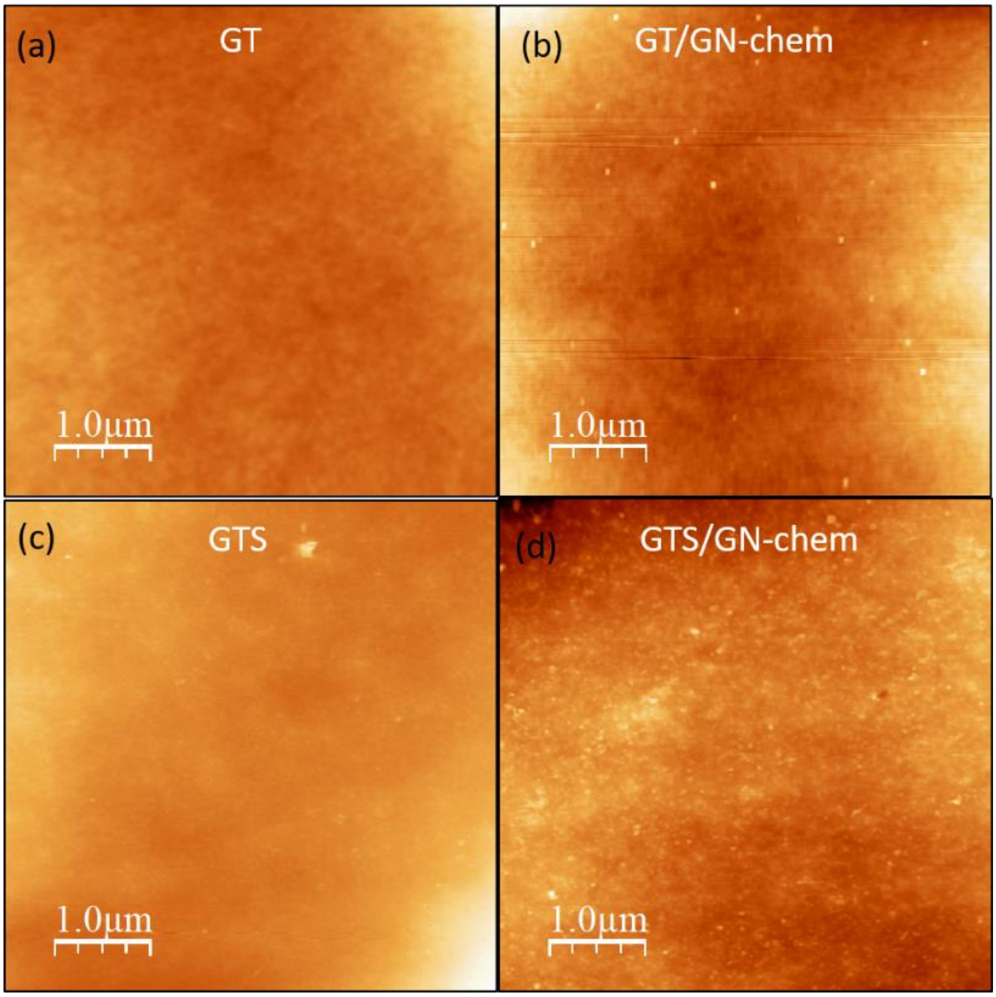

Figure S4. AFM topography images of (**a**) GT, (**b**) GT/GN-chem, (**c**) GTS and (**d**) GTS/GN-chem coatings on aluminum alloy substrates.

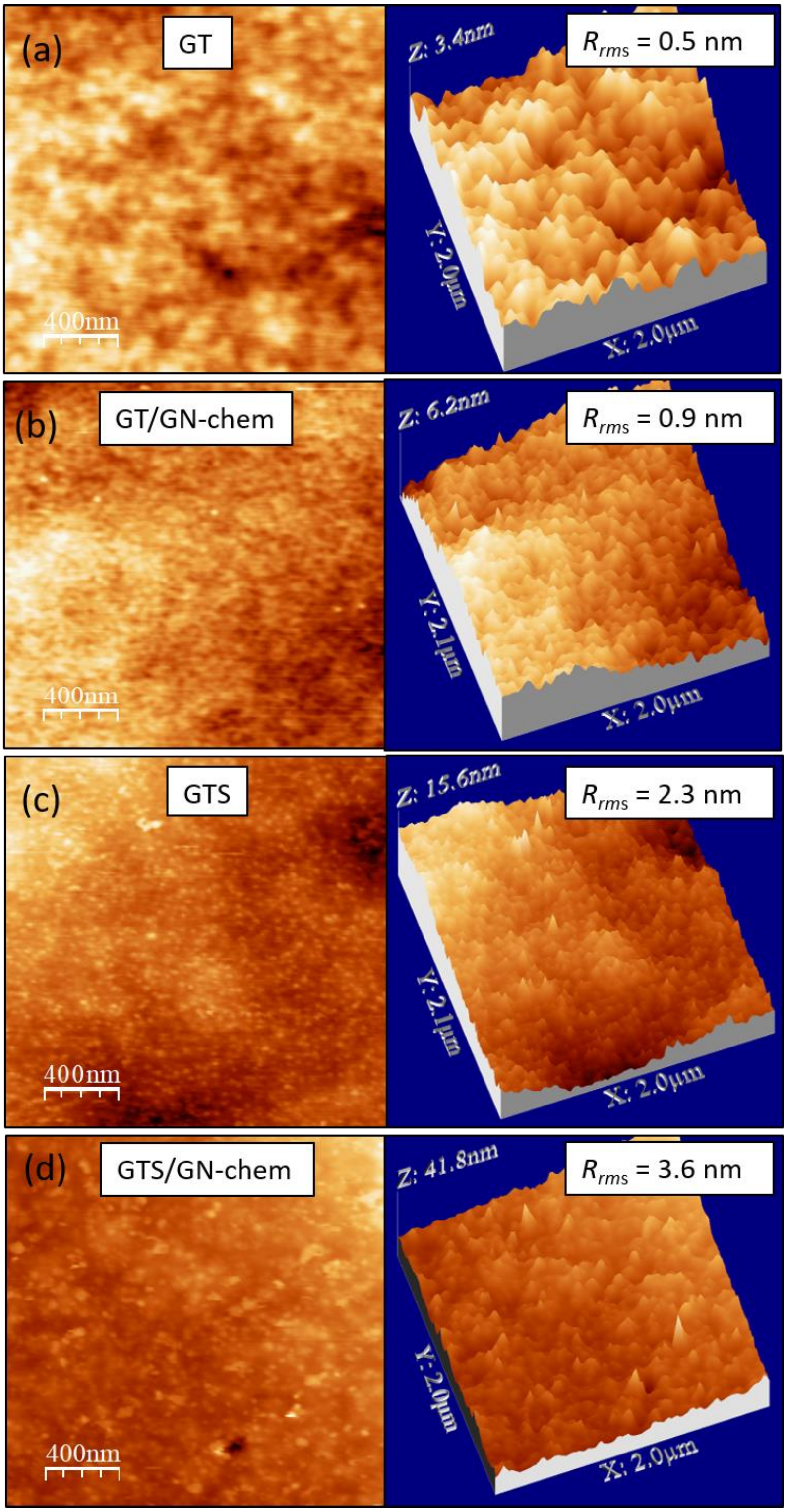

Figure S5. AFM topography 2D images and corresponding 3D views of surface morphology of (**a**) GT, (**b**) GT/GN-chem, (**c**) GTS and (**d**) GTS/GN-chem coatings on aluminum alloy substrates. (XYZ axis values are (**a**) 2.0 µm, 2.0 µm, 3.4 nm (**b**) 2.0 µm, 2.1 µm, 6.2 nm (**c**) 2.0 µm, 2.1 µm, 15.6 nm and (**d**) 2.0 µm, 2.0 µm, 41.8 nm, respectively.

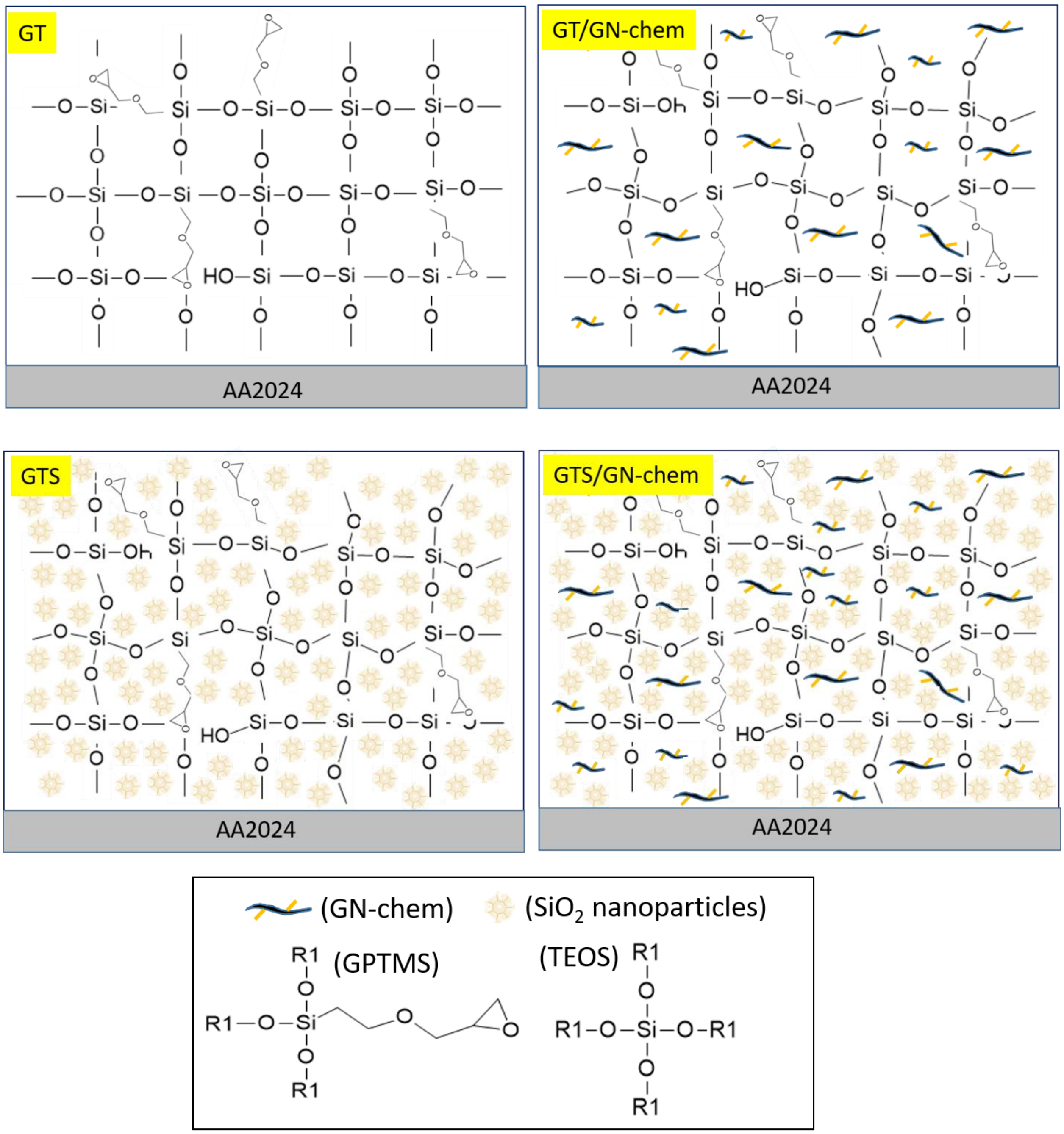

Figure S6. Schematic illustration of GT, GT/GN-chem, GTS and GTS/GN-chem coatings. The structure and the network integrity are shown before and after addition of fillers (GN-chem and SiO_2 nanoparticles).

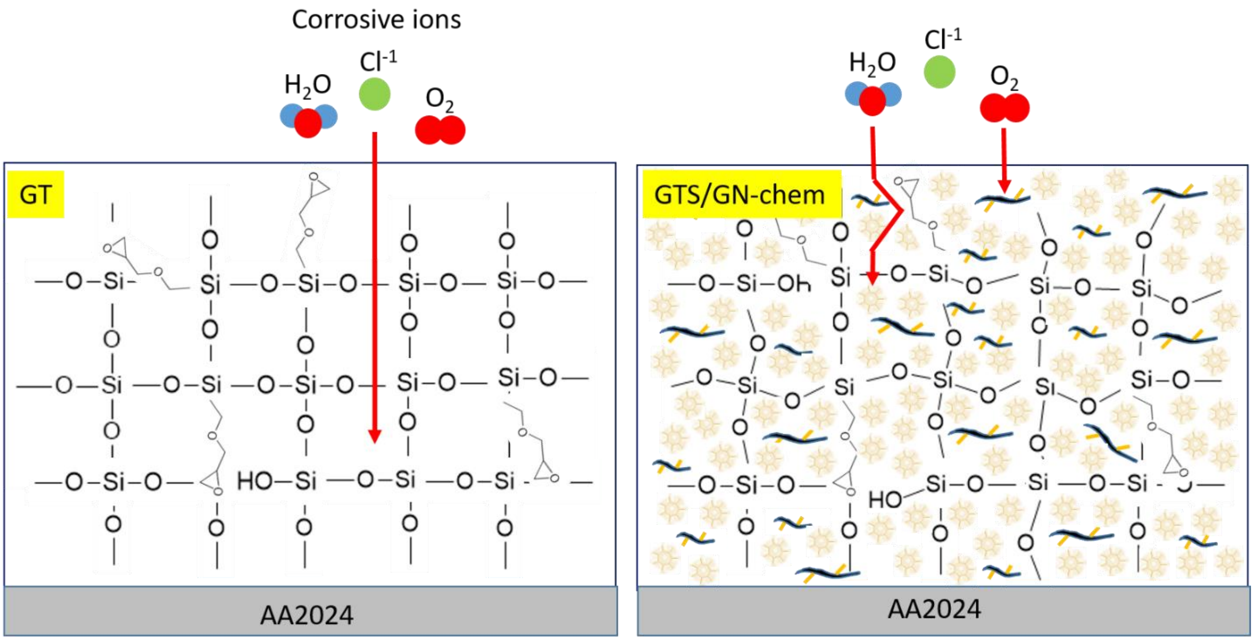

Figure S7. Schematic illustration of the effectiveness of filler addition (GN-chem and SiO_2 nanoparticles) to the coatings in achieving an impermeable barrier against the penetration of corrosive ions. (Red arrows show the track of corrosive ions attacking and penetrating into the coating.).

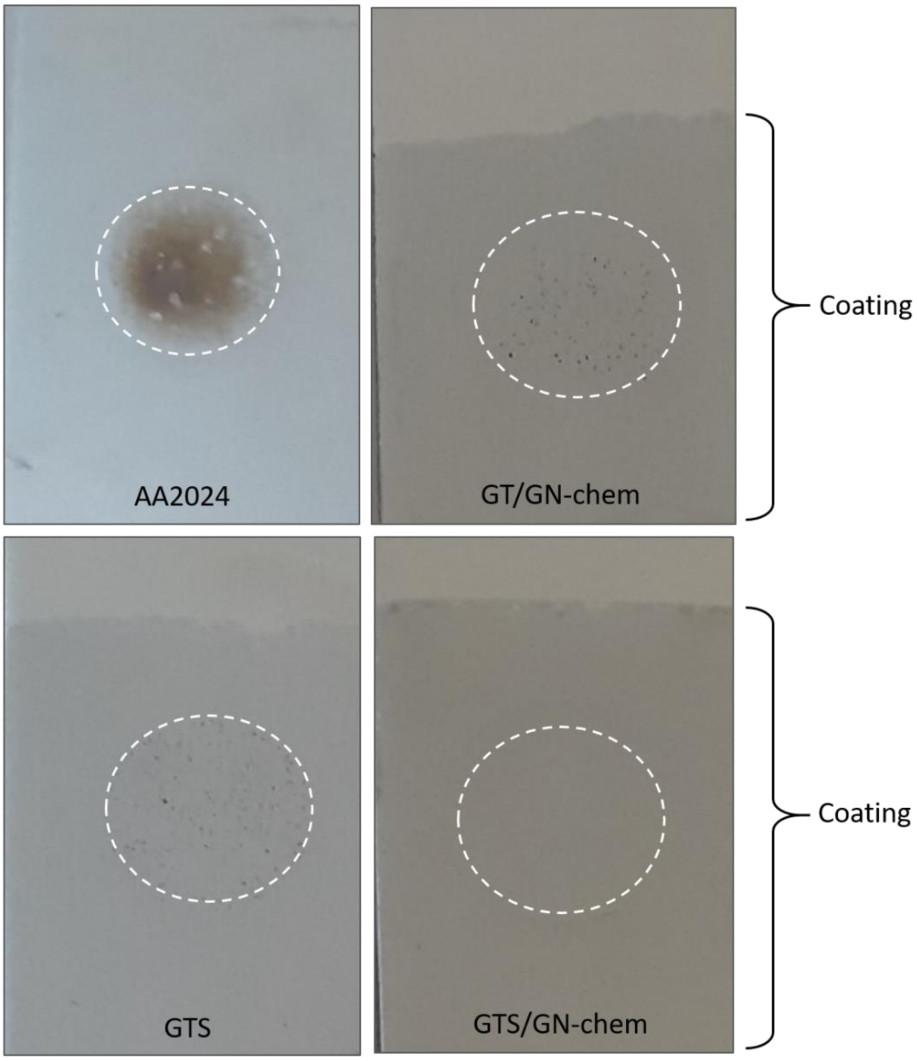

Figure S8. Visual illustration of the surface coating alteration after exposure to the corrosive environment. As evidenced by the image, the lowest surface damage is observed for the GTS/GN-chem coating.

References.

1. Sarkar, S., Niyogi, S., Bekyarova, E. Haddon, R.C. Organometallic chemistry of extended periodic π-electron systems: Hexahapto-chromium complexes of graphene and single-walled carbon nanotubes. *Chem. Sci.* **2011**, *2*, 1326–1333.
2. Zhi, D., Wang, H., Jiang, D., Parkin, I.P., Zhang, X. Reactive silica nanoparticles turn epoxy coating from hydrophilic to super-robust superhydrophobic. *RSC Adv.* **2019**, *9*, 12547–12554.
3. Chandrasekaran, S., Sato, N., Tölle, F., Mülhaupt, R. Fiedler B., Schulte, K. Fracture toughness and failure mechanism of graphene based epoxy composites. *Compos. Sci. Technol.* **2014**, *97*, 90–99.
